# Development an effective system to expression recombinant protein in *E. coli* via comparison and optimization of signal peptides: Expression of *Pseudomonas fluorescens* BJ-10 thermostable lipase as case study

**DOI:** 10.1186/s12934-018-0894-y

**Published:** 2018-03-28

**Authors:** Weiqing Zhang, Jing Lu, Shuwen Zhang, Lu Liu, Xiaoyang Pang, Jiaping Lv

**Affiliations:** Institute of Agro-food Science and Technology, Chinese Academy of Agricultural Sciences (CAAS), Beijing, 100193 China

**Keywords:** Lipase, Signal peptide, Fusion expression, *E. coli*, DsbA

## Abstract

**Background:**

Thermostable lipases from microbial sources have been substantially overexpressed in *E. coli*, however, these enzymes are often produced with low-level enzymatic activity and mainly in the form of inclusion bodies. Several studies have reported that the secretory production of recombinant proteins fused their N-terminus to a signal peptide has been employed to resolve the problem. In general, the feasibility of this approach largely depends on the secretory pathway of signal peptide and the type of target protein to be secreted. This study was performed to compare and optimize signal peptides for efficient secretion of thermostable lipase lipBJ10 from *Pseudomonas fluorescens* BJ-10. Meanwhile, a comparative study between this method and cytoplasmic secretion was implemented in secreting soluble and active lipases.

**Results:**

Fusion expression using six signal peptides, i.e., PelB and five native *E. coli* signal peptides, as fusion partners produced more soluble and functional recombinant lipBJ10 than non-fusion expression. Recombinant lipBJ10, fused to these six diverse signal peptides, was secreted into the periplasm in *E. coli*. The total lipase activity in all cases of fusion expression was higher than those in non-fusion expression. The relative activity peaked when lipBJ10 was fused to DsbA, yielding a value 73.3 times greater than that of the non-fusion protein. When DsbA was used as the fusion partner, the highest activity (265.41 U/ml) was achieved with the least formation of inclusion bodies; the other four *E. coli* signal peptides, to some extent, led to low activity and insoluble inclusion bodies. Therefore, DsbA is the optimal signal peptide partner to fuse with lipBJ10 to efficiently produce soluble and functional protein.

**Conclusion:**

We found that fusing to these signal peptides, especially that of DsbA, can significantly decrease the formation of inclusion bodies and enhance the function and solubility of lipBJ10 compared to non-fusion lipBJ10. Our results reported here can provide a reference for the high-level expression of other lipases with respect to a possible industrial application.

## Background

Lipases (triacylglycerol ester hydrolases, E.C. 3.1.1.3) are serine hydrolases that can catalyze the hydrolysis of long-chain triacylglycerols to liberate long-chain fatty acids and glycerol in aqueous media [1]. Lipases from various sources, especially microbial sources, have been widely used in industrial fields such as organic synthesis and the detergent and dairy industries [2]. Thermophilic and thermostable microbial lipases are currently in great demand because they can be produced at low cost and perform chemical reactions at higher temperatures. Bioprocesses that are performed with thermostable lipases at elevated temperatures have several advantages [2]. However, the application of microbial lipases currently faces two main problems: the vast majority of lipases have poor thermal stability, and the yield of lipase by wild-type strains is low.

In order to solve this problem, gene engineering technologies, because of their ability to highly expression proteins, have been extensively applied to overexpressing thermostable microbial lipases. These lipases can be obtained from both mesophilic and thermophilic organisms, and even psychrophiles have some thermostable enzymes [3]. During recent years, many microbial lipases have been overexpressed in homologous or heterologous hosts such as *E. coli*, yeast or *Bacillus subtilis*. The activity of *Geobacillus thermoleovorans Toshki* lipase, which was overexpressed in *E. coli*, was approximately 4.5-fold higher than in the wild-type strain [4]. Pfeffer et al. reported that thermostable lipase from *Candida antarctica* was functionally expressed in the methylotrophic yeast *Pichia pastoris*, and the lipase concentration reached 0.88 g/l [5]. Although many systems have been successfully applied to the expression of protein, *E. coli* expression system still dominates the bacterial expression systems and remains the preferred system for laboratory investigations and initial development in commercial activities or as a useful benchmark for comparison among various expression platforms [6].

The enteric bacterium *E. coli* is a prokaryotic expression system that is strongly preferred for the following advantages: (1) its ease of genetic manipulation, (2) fast growth rate, (3) well-understood genetics, (4) high yield of recombinant proteins, and (5) low-cost fermentation media. However, the production of proteins by *E. coli* has several disadvantages, including complex downstream processing, weak biological activity, and low product stability and solubility. In addition, recombinant proteins are mainly expressed as inclusion bodies, which are insoluble and inactive, and must be refolded in vitro. Researchers have attempted to overcome this problem by using numerous strategies and technologies such as the optimization of expression conditions [7], the use of fusion tags [8], and the use of periplasmic and even extracellular expression [9].

One of the general methods to enhance the solubility and function of recombinant proteins in *E. coli* is to fuse their N-terminus to a signal peptide, which can lead to the export of the heterologous protein from the cytoplasm into the periplasmic space or the culture medium though the type II secretion system [10]. The type II secretion system that secretes proteins to outside the cell is mediated by periplasmic translocation, which is a two-step process that involves three secretory pathways (the SecB-dependent (Sec), signal recognition particle (SRP), and twin-arginine translocation (TAT) pathways), is widely disseminated among gram-negative bacteria [11]. This genetic technique has assisted in the production of several recombinant proteins by enabling the higher stability of the gene product, correct folding, and improved downstream processing. Several signal peptides of outer membrane proteins (from native and other species) that are transported by the general secretion pathway have been used to efficiently secrete recombinant proteins into the periplasm of *E. coli* [12]. With the N-terminus fusion of DsbA signal peptide, human growth hormone was secreted into periplasm of *E. coli*, reaching a level of 12 µg/ml [13]. Mukherjee et al. demonstrated that *E. coli* cells were capable of secreting 90% of a recombinant single-chain antibody fragment (scFv fused to the PelB signal sequence) into culture supernatant, and the extracellular scFv concentration reached a maximum of 160 mg/l [14].

Despite the successful use of signal peptides of prokaryotic and eukaryotic origins for this purpose [15], the presence of a signal peptide does not always ensure the efficient secretion of recombinant protein into the periplasmic space [16] and the formation of soluble, functional proteins that are correctly folded [17]. Thus, the selection of an optimal signal sequence is important for the efficient secretion of a recombinant protein.

Exogenous lipases that were predominantly produced by psychrotrophic bacteria (primarily *Pseudomonas fluorescens*) found growing in raw milk during refrigerated storage (2–4 °C) maintained 20–30% of their original activity, even under ultrahigh temperature (UHT, 135 °C) for 3–5 s [18]. Psychrotrophic *Pseudomonas fluorescens* BJ-10 was previously isolated in our laboratory from raw milk [19]. The lipase produced by *P. fluorescens* BJ-10 that was isolated from raw milk was still active after normal thermal treatments and even after UHT. The unique characteristics of this protein, lipBJ10, make it a potential biocatalyst in biochemical processes. LipBJ10 was overexpressed in *Escherichia coli* to explore its physical and chemical characteristics and the possibility of its industrial application. To create an efficient recombinant expression system that is propitious to achieving periplasmic expression and the formation of soluble and functional lipBJ10, we constructed six strains that carry different fusion expression plasmids. In this report, we compared the performances of these six strains to determine an optimal signal sequence. In addition, a non-fusion expression plasmid, which lacks the signal sequences of the fusion expression plasmids, was constructed to test and verify whether greater quantities of soluble and functional protein are produced by periplasmic than cytoplasmic expression.

## Results

### Location of recombinant lipBJ10

**Fig. 1:**
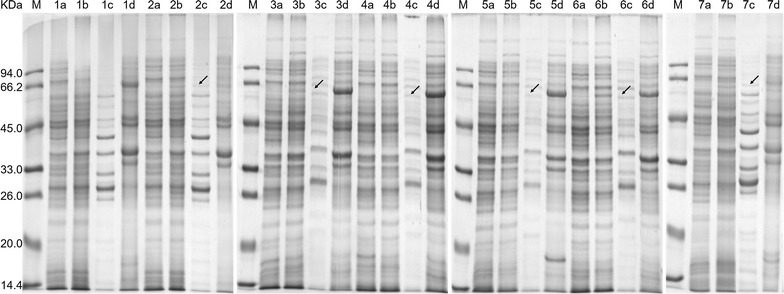
SDS-PAGE analysis of recombinant protein lipBJ10 sample from BL21 (DE3) containing different recombinant plasmid from pET-SigPFL00 to pET-SigPFL06 induced with 0.2 mM IPTG for 40 h at 20 °C. 10 μg of total protein was loaded on a 12% SDS-PAGE gel. Lanes: M, molecular mass standard; 1, S(-)-lipBJ10; 2, DsbA-lipBJ10; 3, FhuD-lipBJ10; 4, MdoD-lipBJ10; 5, OmpA-lipBJ10; 6, YcdO-lipBJ10; 7, PelB-lipBJ10; *a*, uninduced whole-cell sonicated supernatant fraction; *b*, induced whole-cell sonicated supernatant fraction; *c*, periplasmic fraction; *d*, Inclusion bodies. Arrows indicate the position of lipBJ10 in the gel

The vast majority of heterologous proteins destined for periplasmic expression in *E. coli* are synthesized with an amino-terminal signal sequence, 20–30 amino acids in length, that consists of a hydrophobic core followed by a proteolytic cleavage site. In general, the feasibility of fusion expression largely depends on signal peptide, the secretory pathway and the type of target protein to be secreted. The popular PelB signal peptide directs the target recombinant proteins to the *E. coli* periplasm through the SEC pathway. Although the SEC-dependent pathway has been studied in far greater detail and most secretory recombinant protein production strategies use this system, it is often impossible to guarantee that all the recombinant proteins will be translocated by a single targeting pathway [20]. Furthermore, there is no universal signal peptide for a given recombinant protein to guarantee its successful secretion. Thus, the selection of an optimal signal peptide for efficient secretory should be individually researched for each new recombinant protein. Consequently, five different native *E. coli* signal peptides and the PelB signal peptide of three secretory pathways (Table 2) were chosen to target lipBJ10 to the periplasm. To determine whether expression of the protein fused with each signal peptide could facilitate secretion of recombinant lipBJ10 into the periplasmic space, the location of the resulting lipBJ10 was checked by 12% SDS-PAGE. As shown in Fig. 1, a ~ 64.6-kDa protein band, the expected size of lipBJ10, was absent in the supernatants of sonicated uninduced whole cells. However, this band was observed in the periplasmic fraction of all cells that carried the recombinant plasmids pET-SigPFL01 to pET-SigPFL06. The induction of recombinant lipBJ10 (S(-)-PFL) expression in the control strain (carrying pET-SigPFL00), which could be detected by Coomassie straining, was observed only in the IB fraction and not in the supernatant of the sonicated induced whole cells nor in the periplasmic fraction (Fig. 1). The results show that lipBJ10 was secreted to the periplasm in all BL21 (DE3) strains that harbored recombinant plasmids in which the protein was fused to a signal peptide, but lipBJ10 that was expressed without signal predominantly remained in the insoluble form of IBs.

### Analysis of lipBJ10 expression

**Fig. 2:**
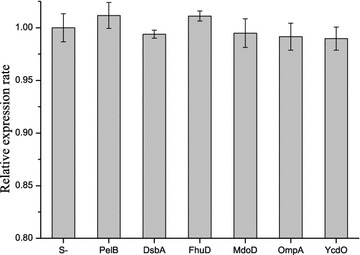
Relative expression level of lipBJ10 in different recombinant strains (from BL21-00 to BL21-06). Data were normalized to the housekeeping gene *E. coli* 16s. Results are shown as relative expression ratios compared with expression in the non-fusion expression strain (the recombinant *E. coli* BL21-00). The results are means ± one standard deviation for three replicates and differences between different strains were statistically significant at a *P* value of 0.05

The expression levels of lipBJ10 in the non-fusion and fusion expression strains (Table 2) were compared by quantitative real-time PCR. No significant difference was observed between the non-fusion and fusion strains in relative expression rate of lipBJ10 (Fig. 2), indicating that fusion expression with these six signal peptides did not change the expression levels of lipBJ10 compared to non-fusion expression under the same conditions. For fusion expression, quantitative real-time PCR analysis revealed a same transcription levels of lipBJ10 between these six-different recombinant *E. coli* strains (from BL21-01 to BL21-06) (Fig. 2).

### Effect on the solubility of lipBJ10

**Fig. 3:**
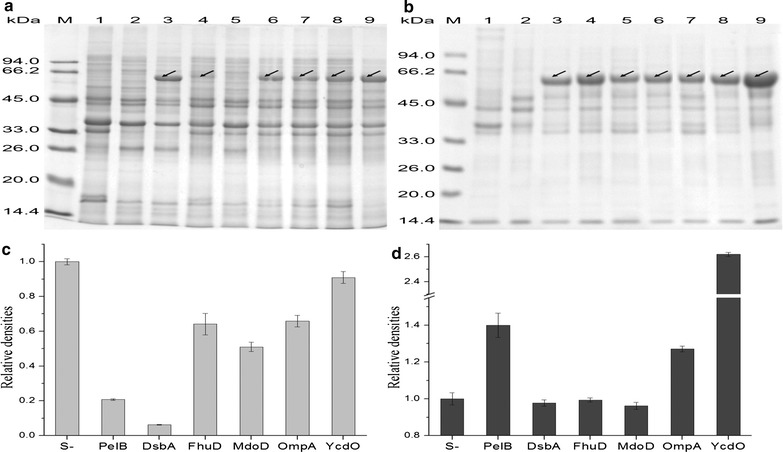
SDS-PAGE analysis of inclusion bodies samples from different BL21 (DE3) strains induced with IPTG for 40 h at 20 °C. Lanes: M, molecular mass standard; 1, BL21 (DE3) (without plasmid); 2, BL21-NULL; 3, BL21-00; 4, BL21-01; 5, BL21-02; 6, BL21-03; 7, BL21-04; 8, BL21-05; 9, BL21-06.10 μg of total protein was loaded on a 12% SDS-PAGE gel. **a** These nine strains were induced with 0.2 mM IPTG for 40 h at 20 °C. **b** These nine strains were induced with 1.0 mM IPTG for 40 h at 20 °C. **c** Quantification of inclusion bodies protein bands in **a** by grayscale. **d** Quantification of inclusion bodies protein bands in **b** by grayscale. The grayscale value of the inclusion bodies protein bands from the non-fusion expression (namely, S-) was set to 1

To study whether expressing lipBJ10 fused with a signal peptide would enhance lipBJ10 solubility, BL21-00 and other recombinant strains were constructed and analyzed (Table 2) under the same fermentation conditions and processing methods. SDS-PAGE analysis shows that the IB content from strains BL21-01 and BL21-02, when the cells were induced by 0.2 mM IPTG, was significantly less than that of BL21-00 and the other fusion expression strains (Fig. 3a). Figure 3c shows that the amount of inclusion bodies, when lipBJ10 was fused to PelB and DsbA, was only 20.8 and 6.1% of that produced by non-fusion expression, respectively. However, some signal-lipBJ10 fusions, such as those formed with signal peptides from OmpA to YcdO, did not significantly decrease the formation of IBs. When lipBJ10 was fused to the other four signals, the formation of IBs were reduced less than 50% (Fig. 3c). IB yields can thus vary greatly based on the diverse signal peptides of the fusions.

Studies have also shown that induction intensity impacts the secretion of recombinant protein. When *E. coli* was induced with high concentrations of IPTG, recombinant protein was mainly found as insoluble IBs and located in the cytoplasm, which is not conducive to the formation of soluble, functional heterologous proteins. We studied whether high concentrations of IPTG would influence the yields of IBs. Figure 3b shows that at high concentrations of IPTG, both non-fusion and fusion protein expression forms large amounts of IBs. Interestingly, we found that the fusions of lipBJ10 with PelB OmpA and Ycdo, under the induction of 1 mM IPTG, resulted in more IBs than non-fusion expression (Fig. 3d). Inducing expression with low concentrations of IPTG, compared with high concentrations, significantly decreases the yield of IBs.

Morphometric analyses, using a Hitachi H-7650B TEM, were performed to further examine the advantages of fusion expression in reducing IB formation and secreting lipBJ10 into the periplasm. Nine different BL21 (DE3) strains (BL21 (DE3) without plasmid and eight strains that were transformed with different plasmids, Table 2) were compared, and BL21 (DE3) and BL21-NULL strains were used as a control group under the same conditions.

**Fig. 4:**
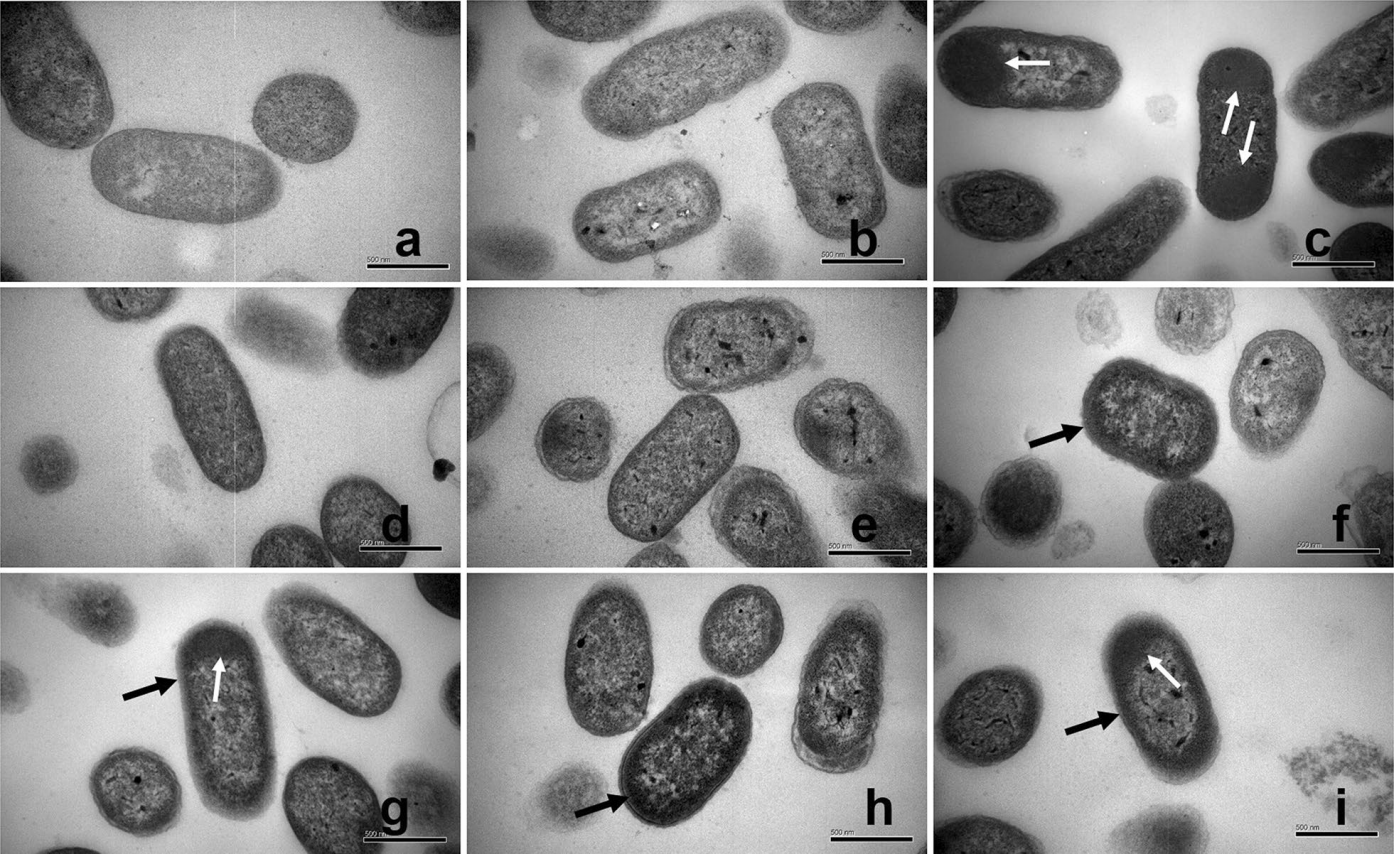
Comparison of Transmission Electron Microscopy of different Cells. The different strains, except BL21 (DE3), were cultivated in LB medium containing Amp (100 mg/ml) and grown at 37 °C until the value of OD_600_ reached 0.8, and then induced with 0.2 mM IPTG for 40 h at 20 °C. The BL21 (DE3) was cultivated in LB medium without Amp. Bar = 500 nm. **a** BL21 (DE3) (without plasmid). **b** BL21-NULL (with empty pET-22b(+)). **c** BL21-00 (with pET-SigPFL00). **d** BL21-01 (DE3) (with pET-SigPFL01). **e** BL21-02 (DE3) (with pET-SigPFL02). **f** BL21-03 (DE3) (with pET-SigPFL03). **g** BL21-04 (DE3) (with pET-SigPFL04). **h** BL21-05 (DE3) (with pET-SigPFL05). **i** BL21-06 (with pET-SigPFL06). White arrow: cytoplasmic inclusion bodies; Black arrow: periplasmic inclusion bodies

Transmission electron micrographs revealed the presence and the amounts of IBs in cells. The TEM image of Fig. 4c clearly shows that a large portion of the cytoplasm was occupied by the newly formed IBs, which appear as electron-dense entities. These forms are barely observed in Fig. 4d, e, in contrast to the control group images (Fig. 4a, b). Figure 4c also demonstrates that cytoplasmic IBs (white arrow) tend to migrate to the poles. This observation is consistent with Rokney’s work [21]. Interestingly, compared with Fig. 4c, the TEM images of Fig. 4f, i clearly demonstrate that most IBs (black arrow) tend to evenly distribute around the interior of the cell rather than at the cell poles. Few IBs were observed in Fig. 4c, d. This phenomenon indicated that expression of the PelB and DsbA fusions led to sufficient solubility.

### Effects on the activity of lipBJ10

**Fig. 5:**
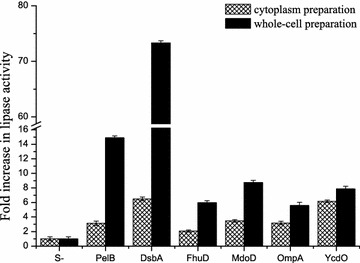
Comparison of the lipase activity of cytoplasmic protein preparation and whole-cell preparation. The different strains (from BL21-00 to BL21-06) were grown at 37 °C until the value of OD_600_ reached 0.8, and then induced with 0.2 mM IPTG for 40 h at 20 °C

BL21 (DE3) *E. coli* strains carrying different recombinant plasmids (Table 2) were incubated under the same conditions, and the BL21-00 strain served as the control group. To study the effects of these signal peptides on recombinant lipBJ10 activity, the supernatants of sonicated cytoplasmic and whole-cell preparations were prepared to measure the lipase activity. The lipase activity in whole-cell preparations of all fusion expression constructs was higher than that of the non-fusion expression (S(-)-lipBJ10); lipase activity peaked when lipBJ10 was fused to DsbA, being 73.3 times greater than that of the non-fusion (Fig. 5). In the control group, the enzyme activity in the cytoplasmic fraction was equal to that in the whole-cell preparation. Lipase activities of the fusions in the whole-cell preparations were significantly higher than those of the fusions in the cytoplasm preparations, and the difference peaked in the DsbA fusion, which was 11.3 times higher (Fig. 5). The results indicate that signal-lipBJ10 fusions are translocated to the periplasm and functionally folded, in contrast to the case when lipBJ10 is expressed without a signal peptide.

### Optimal signal peptide for inducible periplasmic expression of functional lipBJ10

**Fig. 6:**
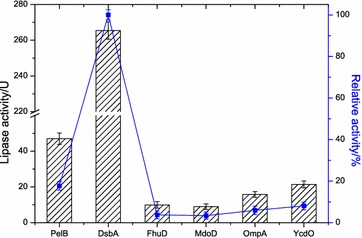
Effect of different signal peptide on the lipase activity of periplasmic protein preparation. The different strains (from BL21-01 to BL21-06) were grown at 37 °C until the value of OD_600_ reached 0.8, and then induced with 0.2 mM IPTG for 40 h at 20 °C

The effects of different signal peptides vary depending on the type of protein to be transported. Strains that carried different fusion expression plasmids (i.e., pET-SigPFL01 to pET-SigPFL06) were all grown at 37 °C until the OD600 value reached 0.8, when they were induced with 0.2 mM IPTG and cultured for 40 h at 20 °C. Lipase activity of proteins in the periplasmic fraction was measured as described in the materials and methods. Strains carrying DsbA and PelB were more effective than four other signal peptides in secreting lipBJ10 into the periplasmic space. When fused to PelB, lipBJ10 activity in the periplasmic space part reached 46.99 U/ml (Fig. 6). However, the activity of lipBJ10 fused to DsbA, which reached 265.41 U/ml, was 5 times greater than that of the PelB fusion (Fig. 6). In addition, the relative activity was just 17.7, 3.7, 3.4, 5.9 and 8.1% of the DsbA fusion activity when lipBJ10 was fused to PelB, FhuD, MdoD, OmpA and YcdO, respectively (Fig. 6). The data suggest that the DsbA signal peptide is the optimal signal peptide for the inducible expression of functional lipBJ10 and its transport into the periplasm.

### Effect of temperature on activity and thermostability of lipBJ10

**Fig. 7:**
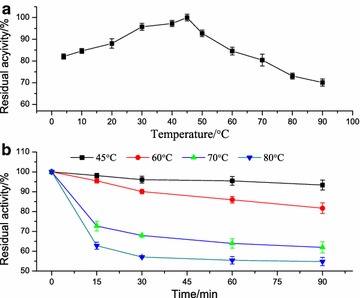
Effect of the temperature on the lipase activity (**a**) and stability (**b**). The highest lipase activity was set to 100%

The temperature at which the activity of lipBJ10 is optimal was observed to be 45 °C, and the enzyme activity was also relatively high in the temperature range from 60 to 90 °C (Fig. 7a). At the high temperature of 60 °C, the relative activity (approximately 84.5%) of this enzyme was similar to the maximum enzyme activity, and it maintained more than 70% of its maximum activity when the temperature was 90 °C. The thermal stability profile of lipBJ10 is shown in Fig. 7b. The enzyme activity changed very little when it spent more time at 45 °C, as its residual activity was greater than 92%. lipBJ10 retained more than 81, 61, and 54% of its residual activity after 90-min incubations at 60, 70, and 80 °C, respectively. Although lipBJ10 displayed relatively lower activity when the temperature was above 60 °C, lipBJ10 was stable, and more than 50% of its original activity was retained at 90 min.

## Discussion

Despite *E. coli* being one of the most frequently used hosts in the high-level production of heterologous proteins for research or commercial purposes, numerous production problems exist in this system. In particular, when proteins are expressed in IBs, they exhibit insolubility and low activity; biologically active proteins can be recovered from IBs, but only through complicated and costly denaturation and refolding processes. In addition, some proteins contain complex disulfide bonds and thus require post-translational modifications to be active. One approach to settling these issues is to have recombinant proteins secreted to the periplasmic space by fusing an appropriate signal sequence to the N-terminus of the target protein gene [20]. However, whether this method is more effective in secreting soluble and active lipases, compared to cytoplasmic secretion, has not been reported. There is thus a need to compare, analyze and illustrate the merits of this method of expressing soluble and functional recombinant proteins. On the other hand, the efficiency of protein secretion varies depending on the host strain, signal sequence, and the type of protein to be secreted [20]. Various signal sequences from bacterial proteins are translocated via different secretion pathways—the Sec (composed of the maltose-binding protein subunit, MalE; pectate lyase subunit, PelB; alkaline phosphatase subunit, PhoA; and outer membrane protein, OmpA), SRP (periplasmic sensory protein, TorT; the subunit of the Tol-Pal cell envelope complex, TolB; and disulfide oxidoreductase, DsbA), and TAT pathways (penicillin acylase, Pac and the subunit of trimethylamine *N*-oxide reductase I, TorA). In general, signal sequences of secretion pathways play an important role in determining protein processing in *E. coli*. Meanwhile, it is necessary to determine the effects of different signal peptides on a given protein (as well as their mutual compatibility) and ultimately screen samples to find an optimal signal sequence.

Several signal sequences, including those from PelB, OmpA, SpA, PhoA, LamB, and DsbA, have been used to efficiently produce recombinant proteins and secrete them to the periplasm in *E. coli* in other studies [20]. Despite many successful examples, this technology still cannot be applied to some proteins, such as human granulocyte-colony stimulating factor (hGCSF) [16]. In this study, all signal-lipBJ10 fusion proteins, i.e., PelB-lipBJ10, DsbA-lipBJ10, FhuD-lipBJ10, MdoD-lipBJ10, OmpA-lipBJ10 and YcdO-lipBJ10, were found to export their lipase into the periplasmic fraction. The current study revealed that fusing lipBJ10 with these seven signal peptides resulted in the expression and secretion of lipBJ10 into the periplasmic space of *E. coli* cells.

The aggregation of recombinant proteins can be diminished by fusing the target protein to a highly soluble fusion partner, such as an affinity tag or a signal peptide [8, 22]. Our study shows that non-fused recombinant lipBJ10 almost solely existed as IBs. In contrast, when lipBJ10 was fused to these six different signal peptides, part of the lipases was secreted into the periplasm and was found in a soluble form. However, the amount of this part of lipases varied according to the kind of signal peptide. When lipBJ10 was expressed with these six signal peptides, the amount of inclusion bodies was 9.1–93.9% less than that produced by non-fusion expression. Upon induction, although the four signal-lipBJ10 fusions other than those of PelB and DsbA still produced some IBs; the activities of sonicated whole cells preparation that contained the fusion proteins were 6.0-, 8.8-, 5.6- and 7.9-fold higher than the activities from cells with non-fusion proteins. The results show that compared with non-fusion protein expression, expression of proteins fused with all six of these different signal peptides reduces IB production and enhances the solubility of lipBJ10. In addition, the decrease in the abundance of IBs was related to the periplasmic expression, and the solubility of the recombinant protein lipBJ10 was enhanced as a result of its fusion to signal peptides. This fusion method, using these six signal peptides as fusion partners, is therefore better at producing soluble and functional recombinant lipBJ10 than non-fusion expression is.

Although many of the protein fusions with signal peptides were very soluble and their production was efficient, the signal peptides are not equally effective as solubility enhancers for improving the solubility of recombinant proteins in vivo. Of all six fusion constructs, the least IB content was clearly formed form the DsbA-lipBJ10 fusion lipBJ10 (Fig. 3a). The TEM image in Fig. 3e shows that there are few IBs within the cells with this fusion protein, while varying extents of IB formation is clearly shown in the TEM images of cells with the other fusion proteins (Fig. 4). In addition, the TEM images from Fig. 4f–i demonstrate that most electron-dense entities (black arrow) tend to evenly distribute around the interior of the cell rather than at the poles. Rokney et al. reported that most IBs were transported to the poles by a specific and energy-dependent process in *E. coli* [21]. A possible cause of this change is that the signal peptide that is fused to the recombinant target protein altered the process. In contrast, Jean-Michel Betton et al. reported that mature protein formed IBs in the periplasm [17]. In this work, we speculated that lipBJ10 proteins fused to a signal peptide other than that of DsbA partially existed in the form of insoluble IBs; the lipase activity of the periplasmic fraction was thus consequently higher when lipBJ10 was fused to DsbA. Accordingly, our findings suggest that DsbA is the optimal signal peptide to enhance the solubility of lipBJ10.

Although lipBJ10 was secreted into the periplasmic space (Fig. 1), enzyme activity varied widely among different fusion proteins. The activity of the DsbA-lipBJ10 fusion in the periplasmic fraction was 5 times higher than that of the corresponding PelB fusion (Fig. 6). This result may be related to the different secretory pathways of PelB (Sec) and DsbA (SRP). Protein export through the Sec pathway occurs post-translationally, whereas export through the SRP pathway is co-translational [23]. For Sec-dependent export to occur, the mature polypeptide must be maintained in an unfolded or perhaps partially folded conformation before being inserted into the SecYEG pore. This insertion is accomplished by interactions with the chaperone SecB or, in some instances, by the actions of DnaK [24]. In contrast, premature folding in the cytoplasm is not an issue for proteins that are exported in an SRP-dependent manner, as protein synthesis is arrested until the ribosome is in contact with the secretion pore [20]. The SRP pathway has an advantage in the secretion of polypeptides that are prone to aggregation in the cytoplasm, which is an issue with proteins that are exported via the post-translational Sec pathway.

The characteristics of the signal peptide also significantly impact the solubility and activity of the recombinant protein. DsbA, a soluble, periplasmic protein containing a C-P-H-G active site embedded in a thioredoxin-like fold, uses its highly reactive Cys30 to promote disulfide transfer in substrate proteins by the formation of mixed disulfide species [25]. It is essential for the function and stability of heterologous proteins to enhance the formation of structural disulfide bonds, as they are conducive to oxidative folding and the activity and stability of many proteins exported from the cytoplasm. However, the *E. coli* cytosol is a rather reducing environment, and disulfide bonds are thus not normally formed there. DsbA is the main introducer of disulfide bonds and has the second highest redox potential among the known TRX-related proteins in the periplasm of *E. coli* [26]. Based on this characteristic, DsbA was widely used in the construction of commercial expression plasmids [27] to enable periplasmic or extracellular secretion [28]. In this work, the highest activity of lipBJ10 was achieved as a result of its fusion to the DsbA signal peptide. This result suggests that lipBJ10 contains a massive number of disulfide bonds and that DsbA catalyzed the formation of the correct disulfide bonds, which is required for the protein to be functional in the periplasm. DsbA was therefore the optimally efficient signal peptide to fuse with lipBJ10 to express soluble and functional lipBJ10 protein.

In addition, formation of IBs in recombinant expression systems is the result of an unbalanced equilibrium between in vivo protein aggregation and solubilization [12]. Large portions of the newly formed lipBJ10 proteins, which were rapidly produced because of a high IPTG concentration (that is, 1 mM), were allocated to IBs in the balancing of aggregation and solubilization. This result suggested that the stress of high concentrations of IPTG was not conducive to the expression of soluble recombinant proteins. Furthermore, IPTG can be toxic to cells at high concentrations [29]. Thus, the stress of high concentrations of IPTG was not conducive to the expression of soluble recombinant protein but might also affect the secretion of proteins into the periplasm. In addition, the fusion expression of lipBJ10 with these six signal peptides did not significantly reduce the formation of inclusion bodies compared with non-fusion expression under the induction of high concentration of IPTG (1 mM). On the contrary, the amount of the inclusion bodies increased 39.0, 27.6 and 161.4% respectively as a result of fusion expression with PelB, OmpA and YcdO. The possible reasons are that the signal-lipBJ10 was expressed at high rates which was not conducive to the folding of protein and that the fusion expression with signal peptide, which increases the molecular weight of the recombinant protein, causes changes in the folding kinetics.

The temperature of optimum activity of the recombinant lipBJ10 was observed to be 45 °C and similar to that of the lipases from *Pseudomonas fluorescens* MTCC 2421 (40 °C) [30] and *Pseudomonas fluorescens* JCM5963 (55 °C) [31]. Although the optimum temperature is below 60 °C, more than 70% of the maximum enzyme activity is maintained in the elevated temperature range of 60–90 °C. On the other hand, lipBJ10 was stable after being incubated at 60 °C for 90 min, with a residual activity greater than 81% (Fig. 7b). Chung et al. reported that the time required for 90% inactivation of a thermostable lipase (the D value) was 4 h at 95 °C and that the increase in temperature that was required to reduce the D value by 90% (the Z_D_ value) was 76 °C [32]. In our study, the D value of lipBJ10 was 72.5 h at 60 °C (data not shown). These results indicate that recombinant lipBJ10 can be used in high-temperature biocatalytic processes, which can surmount certain drawbacks arising from the more stringent requirements for materials, more difficult post-reaction inactivation, and restrictions in the cases of labile substrates or products [2].

## Conclusion

In conclusion, we succeeded in establishing and optimizing an expression system of a heat-resistant lipase from *Pseudomonas fluorescens* BJ-10 in this work. The DsbA signal peptide was determined to be the optimal signal peptide partner to produce soluble, functional, thermostable lipBJ10, which has good prospects for industrial application. More importantly, this work highlights the benefits of periplasmic expression compared with cytoplasmic expression. Meanwhile, our study illustrates the importance of screening signal peptides to determine which one is optimal in the functional expression of target proteins at a high level.

## Methods

### Strains, plasmid, and enzymes

The *P. fluorescens* BJ-10 (CGMCC No.13279) strain used in this study is a psychrophilic strain that was isolated from raw milk. *E. coli* strains DH5α (TIANGEN Biotech (Beijing) Co., Ltd.) and BL21 (DE3) (TIANGEN Biotech (Beijing) Co., Ltd.) were used as cloning and overexpression hosts. The plasmid pET-22b(+) was purchased from Novagen (Madison, WI) and used as the expression vector. DNA polymerase and restriction enzymes *Xho*I, *Eco*RI, and *Nde*I were purchased from NEB (New England Biolabs). The oligonucleotide primers and signal peptides used in this study were synthesized by BGI (Beijing, China) Co., Ltd.

### Construction of lipase expression vector

*Pseudomonas fluorescens* BJ-10 was grown in nutrient broth (10 g/l peptone, 5 g/l NaCl, 3 g/l yeast extract, and 1 g/l glucose, pH 7.0) at 28 °C and 200 rpm for 24 h. The genomic DNA, isolated by using a TIANamp Bacteria DNA Kit (TIANGEN Biotech (Beijing) Co., Ltd.), was used as a template for polymerase chain reaction (PCR) amplification of the lipBJ10 sequence (GenBank Accession No. KY939609). The full-length sequence of lipase gene lipBJ10 was amplified by using primers PFL-22b (Table 1). The PCR product, encoding the mature lipBJ10, and plasmid pET-22b(+) were both digested with restriction enzymes *Nde*I and *Xho*I, and then the fragments were ligated with T4 ligase (TIANGEN Biotech (Beijing) Co., Ltd.) at 16 °C overnight. The resulting plasmid, without a signal peptide, was named pET-SigPFL00.

**Table 1:** Oligonucleotides used in the experiments

Oligonucleotides	Sequence (5′–3′)
PFL-22b (upstream)^a^	CCGCATATGGGTGTCTACGACTACAAAAACC
PFL-22b (downstream)^a^	CCGCTCGAGGGTAATCACAAACGCCTCCG
PFL-SP-22b (upstream)^b^	CCGGAATCCATGGGTGTCTACGACTA
PFL-SP-22b (downstream)^b^	CCGCTCGAGGGTAATCACAAACGCCT
pET-PFL (upstream)^c^	GGGGAATTGTGAGCGGATAAC
pET-PFL (downstream)^c^	TGGCAGCAGCCAACTCAG
16s-reference (upstream)^d^	AATCATCATGCCCCTTATGACC
16s-reference (downstream)^d^	GTTGCAGCCTACAATCCGAAC
PFL-target (upstream)^e^	GGTGGAAGTCCTGGGCAAAT
PFL-target (downstream)^e^	CGCCGATGGAATCAACAA

^a^Primers PFL-22b were used to amplify the full-length sequence of lipase gene lipBJ10, with *Nde*I and *Xho*I restriction sites (underlined), respectively

^b^For fusion with six different signal peptides, primers PFL-SP-22b were used to amplify the full-length sequence of lipase gene lipBJ10, with *Eco*RI and *Xho*I restriction sites (underlined), respectively

^c^Primers pET-PFL were used to verify the sequence of recombinant plasmid

^d^Primers 16s-reference were used to amplify *E. coli* 16s sequence

^e^Primers PFL-target were designed for lipBJ10

For construction of vectors with different signal peptide, a new recombinant plasmid named pET-SigPFL was prepared as follows. The lipBJ10 gene was amplified using synthesized primers PFL-SP-22b (Table 1) to produce an approximately 1848-bp fragment with terminal *Eco*RI and *Xho*I restriction sites. The PCR-synthesized DNA product was cloned into pET-22b(+) that had been digested by restriction enzymes *Eco*RI and *Xho*I. The resulting recombinant plasmid, pET-SigPFL, was transformed into *E. coli* DH5α cells. Recombinants were selected on LB agar plates at 37 °C (1% (w/v) NaCl, 1% (w/v) tryptone, 0.5% (w/v) yeast extract, and 2% (w/v) agar, pH 7.0) containing 100 μg/ml ampicillin. Positive recombinants were inoculated and cultivated in LB liquid medium (1% (w/v) NaCl, 1% (w/v) tryptone, and 0.5% (w/v) yeast extract, and pH 7.0) containing 100 μg/ml ampicillin at 37 °C for 12 h, and the plasmid pET-SigPFL was isolated and then sequence-verified using the primers pET-PFL (Table 1). The plasmid pET-SigPFL was subsequently digested with restriction enzymes *Nde*I and *Eco*RI and then ligated with six different signal peptide nucleotide sequences at 16 °C overnight using T4 ligase. The resulting plasmids, each with different signal peptide, were named pET-SigPFL01, pET-SigPFL02, pET-SigPFL03, pET-SigPFL04, pET-SigPFL05 and pET-SigPFL06 (Table 2). The sequences of all the recombinant plasmids were verified using the primers pET-PFL.

**Table 2:** Nucleotide sequence of signal peptide used in this study and the putative secretory pathway they follow

Strain name	Plasmid name	Putative secretion pathway	Signal peptide name	Signal peptide nucleotide sequence	Recombinant lipBJ10 name
BL21-NULL	pET-22b(+)	–	–	–	–
BL21-00	pET-SigPFL00	–	–	–	S(-)-lipBJ10
BL21-01	pET-SigPFL01	SEC	PelB	ATGAAATACCTGCTGCCGACCGCTGCTGCTGGTCTGCTGCTCCTCGCTGCCCAGCCGGCGATGGCC	PelB-lipBJ10
BL21-02	pET-SigPFL02	SRP	DsbA	ATGAAAAAAATTTGGCTGGCGCTGGCGGGCCTGGTGCTGGCGTTTAGCGCTAGCGCC	DsbA-lipBJ10
BL21-03	pET-SigPFL03	TAT + SEC	FhuD	ATGAGCGGCCTGCCGCTGATTAGCCGCCGCCGCCTGCTGACCGCGATGGCGCTGAGCCCGCTGCTGTGGCAGATGAACACCGCGCATGCC	FhuD-lipBJ10
BL21-04	pET-SigPFL04	TAT + SEC	MdoD	ATGGATCGCCGCCGCTTTATTAAAGGCAGCATGGCGATGGCGGCGGTGTGCGGCACCAGCGGCATTGCTAGCCTGTTTAGCCAGGCGGCGTTTGCC	MdoD-lipBJ10
BL21-05	pET-SigPFL05	SEC	OmpA	ATGAAAAAAACCGCGATTGCGATTGCGGTGGCGCTGGCGGGCTTTGCGACCGTGGCGCAGGCC	OmpA-lipBJ10
BL21-06	pET-SigPFL06	TAT + SEC	YcdO	ATGACCATTAACTTTCGCCGCAACGCGCTGCAGCTGAGCGTGGCGGCGCTGTTTAGCAGCGCGTTTATGGCGAACGCC	YcdO-lipBJ10

### Culture conditions and induction of lipBJ10 expression

Although many recombinant proteins have been successfully produced using *E. coli* as the host, these proteins are often produced in the form of inclusion bodies. In general, lowering the cultivation temperature is an effective strategy to overcome the formation of insoluble and/or nonfunctional inclusion bodies in *E. coli*. Temperature-shift cultivation method has been reported [33, 34]. The whole fermentation process is divided into two stages; that is, the cell growth phage and the protein overexpression phage. The host strain is cultivated at the optimum growth temperature (the cell growth phase) and, subsequently, the cultivation temperature is reduced for the protein expression (the protein production phase) [35]. In the prophase study, we have investigated the effect of different post-induction temperatures on efficient expression of our target protein lipBJ10. The results showed that the optimum post-induction temperature was 20 °C (data not shown).

All seven recombinant plasmids and empty plasmid pET-22b(+) (Table 2) were transformed into BL21 (DE3) resulting in eight different strains. These eight strains were cultivated in 20 ml of LB liquid medium containing 100 µg/ml ampicillin at 37 °C and 200 rpm. The control BL21 (DE3) strain was also grown in 20 ml of LB liquid medium but without ampicillin at 37 °C and 200 rpm. When the optical density (OD) at 600 nm reached 1.0–1.2, isopropyl-β-D-thiogalactoside (IPTG) was added to a final concentration of 0.2 mM or 1.0 mM to induce lipBJ10 expression, and the temperature was decreased to 20 °C.

### Periplasmic protein preparation

After 40 h of induction at 20 °C, the cultured cells were harvested by centrifugation at 10,000*g* and 4 °C for 5 min. The periplasmic fraction was prepared by modifying a previously described method [36]. The harvested cells were resuspended in 1 ml of ice-cold periplasmic extraction buffer I (20% (w/v) sucrose, 100 mM Tris–HCl, pH 8.0). The mix was incubated on ice for 30 min. The cells were pelleted at 10,000*g* and 4 °C for 10 min. The supernatant, i.e., the periplasmic fraction, was removed with a pipette. The remaining pellet fraction was resuspended in 1 ml of periplasmic extraction buffer II (50 mM MgCl_2_) and incubated on ice for 20 min. The cells were pelleted at 10,000*g* and 4 °C for 10 min. The supernatant, which was the osmotic shock fraction, was recovered and combined with the periplasmic fraction. The mixture represented the periplasmic fraction of the cells.

### Cytoplasmic supernatant and inclusion bodies (IBs)

The remaining pellet, without its periplasmic fraction, was resuspended in 3 ml of lysis buffer (50 mM Tris–HCl, pH 8.0) and sonicated to completely disrupt the cells. The IBs were pelleted by centrifugation at 12,000*g* for 15 min, and the supernatant that was collected was denoted the cytoplasmic supernatant. The IBs were prepared as follows. The IBs were washed with 3 ml of lysis buffer containing 2% Triton X-100 and pelleted again by centrifugation at 12,000*g* for 15 min. The pellet was subsequently resuspended in 1 ml of denaturation buffer (50 mM Tris–HCl, 8 M urea, and 20 mM β-mercaptoethanol, pH 8.0), and the supernatant that was collected was denoted the solubilized IBs.

### Lipase activity

Lipase activity assays were performed by means of the modified colorimetric method, using *p*-nitrophenyl caprylate as the substrate. Briefly, 200 μl of buffer (50 mM Tris–HCl, pH 8.0), 20 μl of substrate solution (5 mM *p*-nitrophenyl caprylate dissolved in acetonitrile), and 20 μl of sample were added. After the mixture was stirred, it was incubated at 45 °C for 20 min and then its absorption was measured at 405 nm using a Microplate reader (BIO-RAD Model 680) purchased from Bio-Rad Laboratories, Inc. (Hercules, California, USA). One unit of lipase activity (U) was defined as the release of 1 μmol of *p*-nitrophenol per min.

### Protein quantitation and SDS-PAGE

Protein concentrations were measured using the bicinchoninic acid (BCA) assay [37] with bovine serum albumin (BSA) as standard. Sodium dodecyl sulfate–polyacrylamide gel electrophoresis (SDS-PAGE) was performed according to the method of Laemmli (1970) [38] using 12% polyacrylamide separation and 5% polyacrylamide stacking gels on a vertical mini-gel apparatus (Bio-Rad) at 120 V for 1.5 h. The gel was stained with Coomassie brilliant blue R-250 (Bio-Rad) to detect protein and analyzed by using a gel imaging analysis system (AlphaEaseFC). The protein bands were relatively quantified by grayscale scanning with the system self-contained software.

### Quantitative real-time PCR analysis

Total RNA was extracted from cells using RNAprep pure Cell/Bacteria Kit (TIANGEN Biotech, Beijing, China) according to the manufacturer’s protocol. Reverse transcription was performed with FastQuant RT Kit (TIANGEN Biotech, Beijing, China) following the manufacturer’s instructions. Quantitative real-time PCR was conducted using SuperReal PreMix Plus (SYBR GREEN) (TIANGEN Biotech, Beijing, China), and fluorescence amplification was carried out using an ABI 7500 Fast Real-time PCR System (Applied Biosystems, Foster City, CA, USA). Oligonucleotide primers used for quantitative real-time PCR analysis were listed in Table 1. The *E. coli* 16s mRNA was used as the internal control. The relative quantity of lipBJ10 gene expression was calculated using the comparative cycle threshold (∆∆Ct) method [39].

### Transmission electron microscope

Morphometric analyses were performed using a Hitachi H-7650B (Hitachi, Japan) transmission electron microscope (TEM) [40].

### Effect of temperature on activity and thermostability of lipBJ10

The effect of temperature on lipBJ10 activity was assayed in the range from 4 to 90 °C in Tris–HCl buffer (50 mM, pH 8.0). The optimal activity of lipBJ10 was found to occur at pH 8.0 (data not shown). The residual lipase activity was calculated with the enzyme activity at the optimum temperature set to 100%. The thermostability of lipBJ10 was investigated by preincubating the enzyme solution at various temperatures (45–80 °C) for 15, 30, 60, and 90 min. The enzyme’s residual activity was measured in Tris–HCl buffer (50 mM, pH 8.0) at 45 °C under standard assay procedures. To investigate the properties of the lipBJ10 protein that was overexpressed by *E. coli* BL21-02, the recombinant lipases that were extracted from the periplasmic space were purified by using nickel affinity chromatography (Ni–NTA) with an AKTA purifier system to exploit the histidine tag [36].

## References

[1] Schmid RD, Verger R (2010). Lipases: interfacial enzymes with attractive applications. Angew Chem Int Ed.

[2] Hasan F, Shah AA, Hameed A (2006). Industrial applications of microbial lipases. Enzyme Microb Technol.

[3] Demirjian DC, MoríS-Varas F, Cassidy CS (2001). Enzymes from extremophiles. Curr Opin Chem Biol.

[4] Abdel-Fattah YR, Gaballa AA (2008). Identification and over-expression of a thermostable lipase from *Geobacillus thermoleovorans* Toshki in *Escherichia coli*. Microbiol Res.

[5] Pfeffer J, Richter S, Nieveler J, Hansen CE, Rhlid RB, Schmid RD, Rusnak M (2006). High yield expression of lipase A from *Candida antarctica* in the methylotrophic yeast *Pichia pastoris* and its purification and characterisation. Appl Microbiol Biotechnol.

[6] Chen R (2012). Bacterial expression systems for recombinant protein production: E. coli and beyond. Biotechnol Adv.

[7] Hoffmann F, Heuvel JVD, Zidek N, Rinas U (2004). Minimizing inclusion body formation during recombinant protein production in *Escherichia coli* at bench and pilot plant scale. Enzyme Microb Technol.

[8] Esposito D, Chatterjee DK (2006). Enhancement of soluble protein expression through the use of fusion tags. Curr Opin Biotechnol.

[9] Shokri A, Sandén AM, Larsson G (2003). Cell and process design for targeting of recombinant protein into the culture medium of *Escherichia coli*. Appl Microbiol Biotechnol..

[10] Jermy A (2009). Bacterial secretion turning the cogs in type VI secretion. Nat Rev Microbiol.

[11] Johnson TL, Abendroth J, Hol WGJ, Sandkvist M (2006). Type II secretion: from structure to function. FEMS Microbiol Lett.

[12] Sørensen HP, Mortensen KK (2005). Advanced genetic strategies for recombinant protein expression in *Escherichia coli*. J Biotechnol.

[13] Soares CR, Gomide FI, Ueda EK, Bartolini P (2003). Periplasmic expression of human growth hormone via plasmid vectors containing the lambdaPL promoter: use of HPLC for product quantification. Protein Eng.

[14] Mukherjee KJ, Rowe DC, Watkins NA, Summers DK (2004). Studies of single-chain antibody expression in quiescent *Escherichia coli*. Appl Environ Microbiol.

[15] Makrides SC (1996). Strategies for achieving high-level expression of genes in *Escherichia coli*. Microbiol Rev.

[16] Chung BH, Sohn MJ, Oh SW, Park US, Poo H, Kim BS, Yu MJ, Lee YI (1998). Overproduction of human granulocyte-colony stimulating factor fused to the pelB signal peptide in *Escherichia coli*. J Ferment Bioeng.

[17] Betton JM, Hofnung M (1996). Folding of a mutant maltose-binding protein of *Escherichia coli* which forms inclusion bodies. J Biol Chem.

[18] Deeth HC, FitzGerald CH (2006). Lipolytic enzymes and hydrolytic rancidity. Adv Dairy Chem.

[19] Zhang S, Lv J (2014). Purification and properties of heat-stable extracellular protease from *Pseudomonads fluorescens* BJ-10. J Food Sci Technol.

[20] Mergulhão FJ, Summers DK, Monteiro GA (2005). Recombinant protein secretion in *Escherichia coli*. Biotechnol Adv.

[21] Rokney A, Shagan M, Kessel M, Smith Y, Rosenshine I, Oppenheim AB (2009). *E. coli* transports aggregated proteins to the poles by a specific and energy-dependent process. J Mol Biol.

[22] Hannig G, Makrides SC (1998). Strategies for optimizing heterologous protein expression in *Escherichia coli*. Trends Biotechnol.

[23] Georgiou G, Segatori L (2005). Preparative expression of secreted proteins in bacteria: status report and future prospects. Curr Opin Biotechnol.

[24] Mujacic M, Bader MW, Baneyx F (2004). *Escherichia coli* Hsp31 functions as a holding chaperone that cooperates with the DnaK-DnaJ-GrpE system in the management of protein misfolding under severe stress conditions. Mol Microbiol.

[25] Baneyx F, Mujacic M (2004). Recombinant protein folding and misfolding in *Escherichia coli*. Nat Biotechnol..

[26] Ito K, Inaba K (2008). The disulfide bond formation (Dsb) system. Curr Opin Struct Biol.

[27] Kurokawa YS-K, Yanagi HT-S, Yura TK-S. DsbA/DsbB/DsbC/DsbD expression plasmid. US Patent 6673569; 2004.

[28] Sun XW, Wang XH, Yao YB (2014). Co-expression of Dsb proteins enables soluble expression of a single-chain variable fragment (scFv) against human type 1 insulin-like growth factor receptor (IGF-1R) in *E. coli*. World J Microbiol Biotechnol.

[29] Pan H, Xie Z, Bao W, Zhang J (2008). Optimization of culture conditions to enhance cis-epoxy succinate hydrolase production in *Escherichia coli* by response surface methodology. Biochem Eng J.

[30] Chakraborty K, Paulraj R (2009). Purification and biochemical characterization of an extracellular lipase from *Pseudomonas fluorescens* MTCC 2421. J Agric Food Chem.

[31] Zhang A, Gao R, Diao N, Xie G, Gao G, Cao S (2009). Cloning, expression and characterization of an organic solvent tolerant lipase from *Pseudomonas fluorescens* JCM5963. J Mol Catal B Enzym.

[32] Chung GH, Lee YP, Yoo OJ, Rhee JS (1991). Overexpression of a thermostable lipase gene from *Pseudomonas fluorescens* in *Escherichia coli*. Appl Microbiol Biotechnol.

[33] Baedeker M, Schulz GE (1999). Overexpression of a designed 2.2 kb gene of eukaryotic phenylalanine ammonia-lyase in *Escherichia coli*. FEBS Lett.

[34] Hoshino K, Itoh K, Masubuchi A, Adachi M, Asakawa T, Watanabe N, Kosaka T, Tanaka Y (2007). Cloning, expression, and characterization of male cynomolgus monkey liver aldehyde oxidase. Biol Pharm Bull.

[35] Semba H, Ichige E, Imanaka T, Atomi H, Aoyagi H (2008). Efficient production of active form recombinant cassava hydroxynitrile lyase using Escherichia coli in low-temperature culture. Appl Microbiol Biotechnol..

[36] Rouet R, Lowe D, Dudgeon K, Roome B, Schofield P, Langley D, Andrews J, Whitfeld P, Jermutus L, Christ D (2012). Expression of high-affinity human antibody fragments in bacteria. Nat Protoc.

[37] Walker JM (1994). The bicinchoninic acid (BCA) assay for protein quantitation. Methods Mol Biol.

[38] Laemmeli UK (1970). Cleavage of structural proteins during the assembly of the head to bacteriophage T4. Nature..

[39] Livak KJ, Schmittgen TD (2001). Analysis of relative gene expression data using real-time quantitative PCR and the 2(−ΔΔC(T)) method. Methods.

[40] Ono B, Kubota M, Kimiduka H, Kawaminami H, Ueto T, Yokosawa S, Iseda M, Yamamoto Y, Murakami Y, Yokota S (2006). Production of a polymer-forming fusion protein in *Escherichia coli* strain BL21. Biosci Biotechnol Biochem.

